# Ubiquitin-conjugating enzyme E2T (UBE2T) and denticleless protein homolog (DTL) are linked to poor outcome in breast and lung cancers

**DOI:** 10.1038/s41598-017-17836-7

**Published:** 2017-12-13

**Authors:** Javier Perez-Peña, Verónica Corrales-Sánchez, Eitan Amir, Atanasio Pandiella, Alberto Ocana

**Affiliations:** 1 Translational Research Unit, Albacete University Hospital and Centro Regional de Investigaciones Biomedicas (CRIB) and CIBERONC, Castilla La Mancha University, Albacete, Spain; 2 Division of Medical Oncology and Hematology, Princess Margaret Cancer Centre, University of Toronto, Toronto, Canada; 3 Cancer Research Center and CIBERONC, CSIC, Salamanca, Spain

## Abstract

Protein ubiquitination and degradation represent druggable vulnerabilities of cancer cells. We used gene expression and functional annotation analyses to identify genes in the ubiquitin pathway which are differentially expressed between normal breast and basal-like tumors. With this approach we identified 16 ubiquitin related genes overexpressed in basal-like breast cancers compared with normal breast. We then explored the association between these genes and outcomes using the KMPlotter online tool. Two genes, the ubiquitin-conjugating enzyme E2T (UBE2T) and the denticleless protein homolog (DTL) were overexpressed and linked with detrimental outcome in basal-like and luminal breast cancer patients. Furthermore, we found that UBE2T and DTL were amplified in around 12% of breast tumors based on data contained at cBioportal. In non-small cell lung adenocarcinomas, UBE2T and DTL were also amplified in around 7% of cases and linked with disease recurrence after surgical resection. No significant molecular alterations or a clear trend for clinical outcome was observed for these genes in ovarian serous cystadenocarcinoma, esophagus-stomach cancer or non-small squamous cell carcinoma. Our data suggest that UBE2T and DTL may have a role in the pathophysiology of breast and lung tumors, opening avenues for future clinical evaluation of agents targeting those proteins or their pathways.

## Introduction

The identification of oncogenic vulnerabilities based on druggable genomic modifications has permitted the development of therapeutic agents^1,2^. Examples include agents such as trastuzumab or lapatinib that target HER2, or B-Raf inhibitors like vemurafenib in melanoma for patients with mutations in that gene^1,2^.

Targeting non-oncogenic alterations can also result in clinical benefit in certain malignant cells that depend on functions that are more relevant for transformed cells when compared with non-transformed ones. For example the inhibition of the proteasome with therapeutic agents like bortezomib or the more novel compound carfilzomib has reached the clinical setting in multiple myeloma^3,4^. Similarly targeting protein folding with agents against Heat Shock Proteins (HSP) is currently in clinical evaluation^5^.

Ubiquitination is a fundamental process in protein degradation^6^. In this process different steps and enzymes are required including ubiquitin activating enzymes (E1), ubiquiting conjugating enzymes (E2) and ubiquitin ligases (E3)^7,8^. Targeting enzymes involved in this process is challenging as different ubiquitin proteins have been described and they also participate in a wide range of different cellular functions^6,8^, including regulation of cell cycle and DNA repair, in which by increasing the degradation of proteins involved in these pathways, they can facilitate tumor progression and metastasis^7,9–11^.

With the purpose of exploring the clinical relevance of genes involved in ubiquitination, we performed gene expression analyses to identify deregulated components of this machinery in several tumors^12,13^.

We identified the ubiquitin-conjugating enzyme E2T (UBE2T) and the denticleless protein homolog (DTL) as upregulated in basal-like breast tumors and amplified in breast cancer. When studied in other solid tumors we also observed an amplification of these proteins in non-small cell lung adenocarcinomas. In basal-like, luminal breast and non-small cell lung adenocarcinomas upregulation of these genes were associated with detrimental outcome.

## Results

### The ubiquitin-conjugating enzyme E2T (UBE2T) and the denticleless protein homolog (DTL) are overexpressed and linked with detrimental outcome

**Figure 1:**
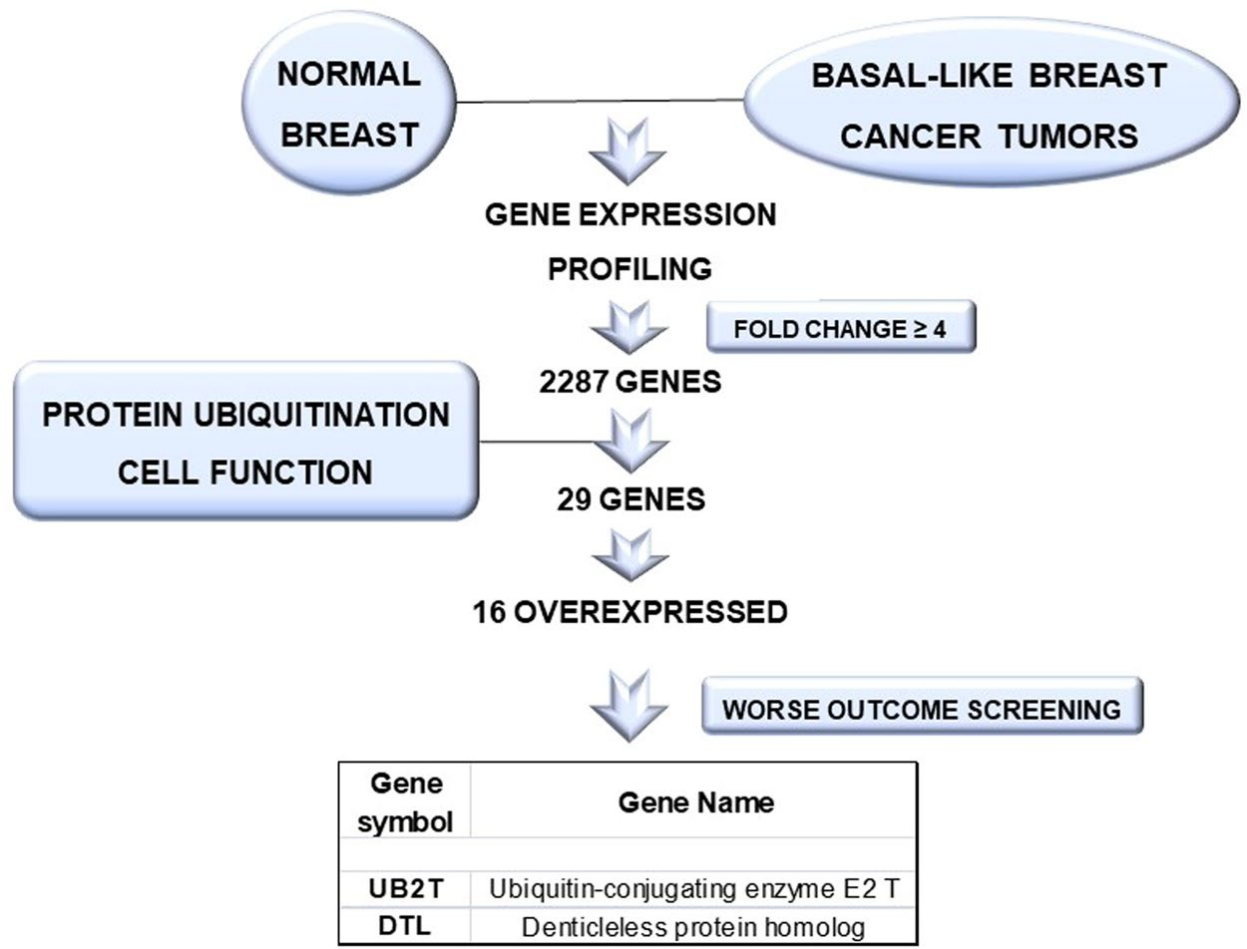
Identification of genes associated with detrimental outcome in Basal-like tumors. Transcriptomic expression analyses among normal breast and Basal-like cancers, with the selection of deregulated genes with more than ≥4 fold change included in the protein ubiquitination cell function. These analysis were performed as described in material and methods. Outcome screening for detrimental relapse free survival using the KM Plotter online tool as described in material and methods.

**Figure 2:**
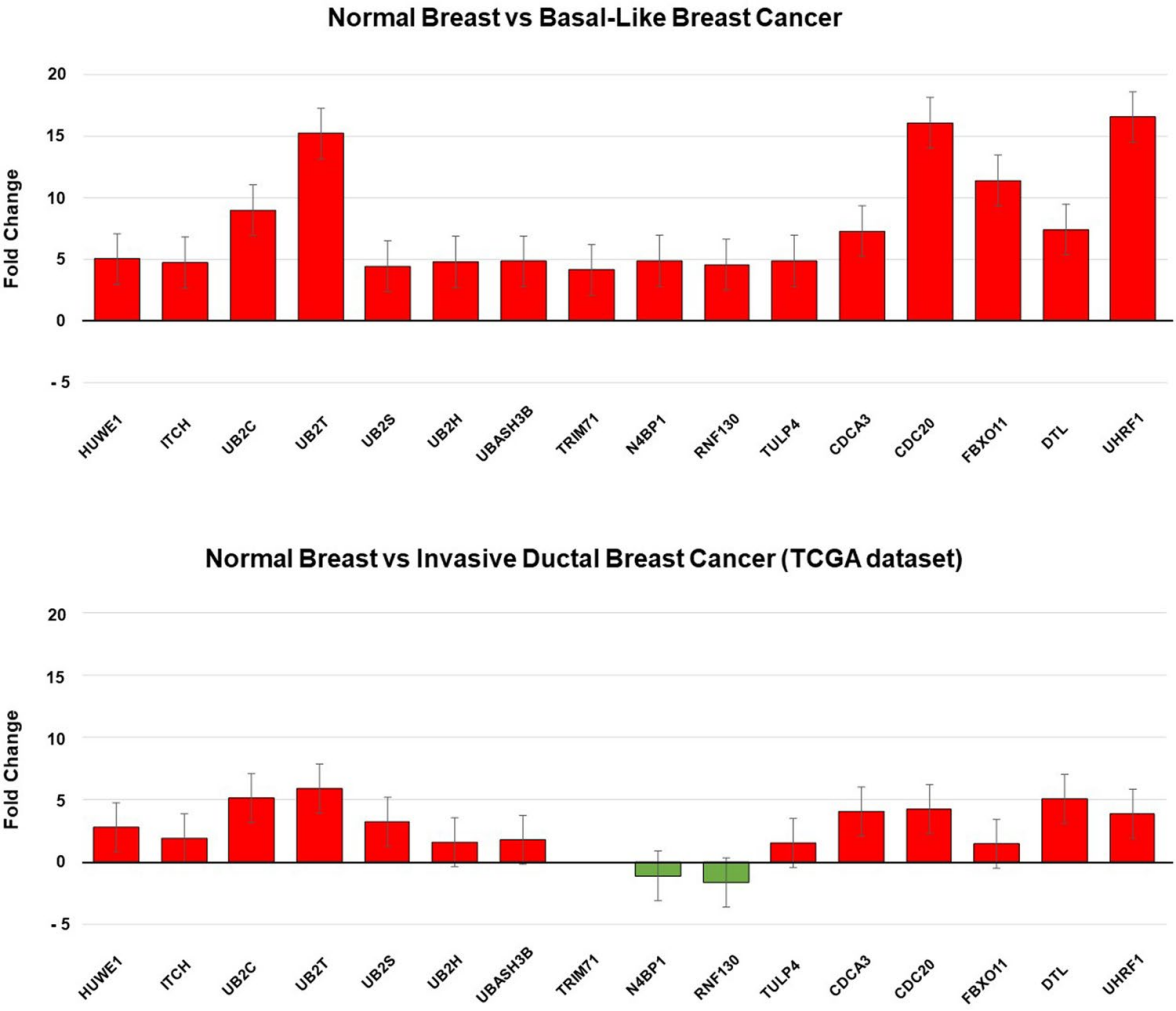
Bar graph showing fold change expression values of the identified genes using the study dataset (GDS2250), upper panel, and data from TCGA, lower pannel. The 95% confidence interval is also displayed.

2287 genes with a minimum 4-fold differential expression value were selected when comparing basal-like tumors with normal breast samples (GEO DataSet accession number: GDS2250). As the hypothesis was to identify those genes linked with protein ubiquitination, only 29 genes were eligible for analysis (Fig. 1). By using Gene Set Enrichment Analysis (GSEA), we confirmed an enrichment of functions linked with ubiquitination and proteasome degradation (Supplementary Figure 1). Within the selected group, 16 genes were overexpressed, and the upregulation of these genes was independently confirmed using the cancer genome atlas (TCGA) database (Fig. 2).

**Figure 3:**
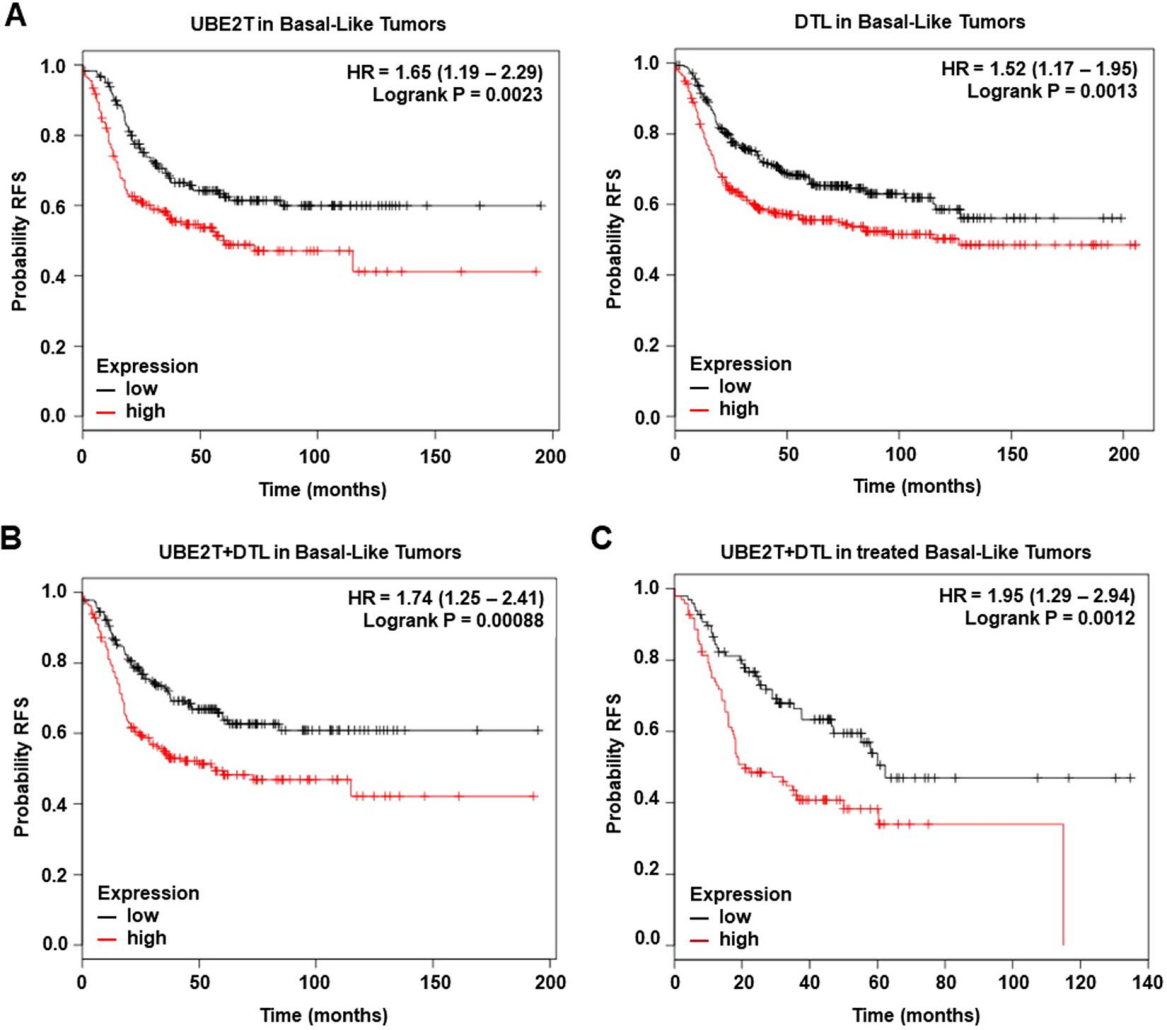
(**A**) Association of UBE2T and DTL individually with relapse free survival in Basal-like tumors. (**B**) Association of the combined analyses of UBE2T and DTL with relapse free survival in Basal-like tumors. (**C**) Association of the combined analyses of UBE2T and DTL with relapse free survival in treated patients with Basal-like tumors.

Using the online tool KM plotter, we identified genes that were associated with detrimental outcome in breast basal-like tumors. Among the 16 genes identified, only two genes, *UBE2T* and *DTL*, were associated with poor relapse free survival (RFS) in the basal subtype (Fig. 3A).

Table 1 describes the function of these genes. UBE2T is an E2 ubiquitin-conjugating enzyme that catalyzed monoubiquitination of several proteins as BRCA1 and FANCD2, both implicated in DNA damage response. DTL is the substrate-specific adapter of the DCX complex, an E3 ubiquitin-protein ligase complex that mediates polyubiquitination of many proteins involved in cell cycle control and DNA damage response. In addition, Supplementary Table 1 describes the biological functions of the remaining 14 identified genes.

**Table 1:** Biological functions of UBE2T and DTL.

Gene name	Gene symbol	Function
Ubiquitin-conjugating enzyme E2T	UBE2T	Accepts ubiquitin from the E1 complex and catalyzes its covalent attachment to other proteins. Catalyzes monoubiquitination. Involved in mitomycin-C (MMC)-induced DNA repair: acts as a specific E2 ubiquitin-conjugating enzyme for the Fanconi anemia complex by associating with E3 ubiquitin-protein ligase FANCL and catalyzing monoubiquitination of FANCD2, a key step in the DNA damage pathway. Also mediates monoubiquitination of FANCL and FANCI. May contribute to ubiquitination and degradation of BRCA1. *In vitro* able to promote polyubiquitination using all 7 ubiquitin Lys residues, but may prefer ‘Lys-11’-, ‘Lys-27’-, ‘Lys-48’- and ‘Lys-63’-linked polyubiquitination.
Denticleless protein homolog	DTL	Substrate-specific adapter of a DCX (DDB1-CUL4-X-box) E3 ubiquitin-protein ligase complex required for cell cycle control, DNA damage response and translesion DNA synthesis. The DCX(DTL) complex, also named CRL4(CDT2) complex, mediates the polyubiquitination and subsequent degradation of CDT1, CDKN1A/p21(CIP1), FBXO18/FBH1 and KMT5A. CDT1 degradation in response to DNA damage is necessary to ensure proper cell cycle regulation of DNA replication. CDKN1A/p21(CIP1) degradation during S phase or following UV irradiation is essential to control replication licensing. KMT5A degradation is also important for a proper regulation of mechanisms such as TGF-beta signaling, cell cycle progression, DNA repair and cell migration. Most substrates require their interaction with PCNA for their polyubiquitination: substrates interact with PCNA via their PIP-box, and those containing the ‘K + 4’ motif in the PIP box, recruit the DCX(DTL) complex, leading to their degradation. In undamaged proliferating cells, the DCX(DTL) complex also promotes the ‘Lys-164’ monoubiquitination of PCNA, thereby being involved in PCNA-dependent translesion DNA synthesis.

**Figure 4:**
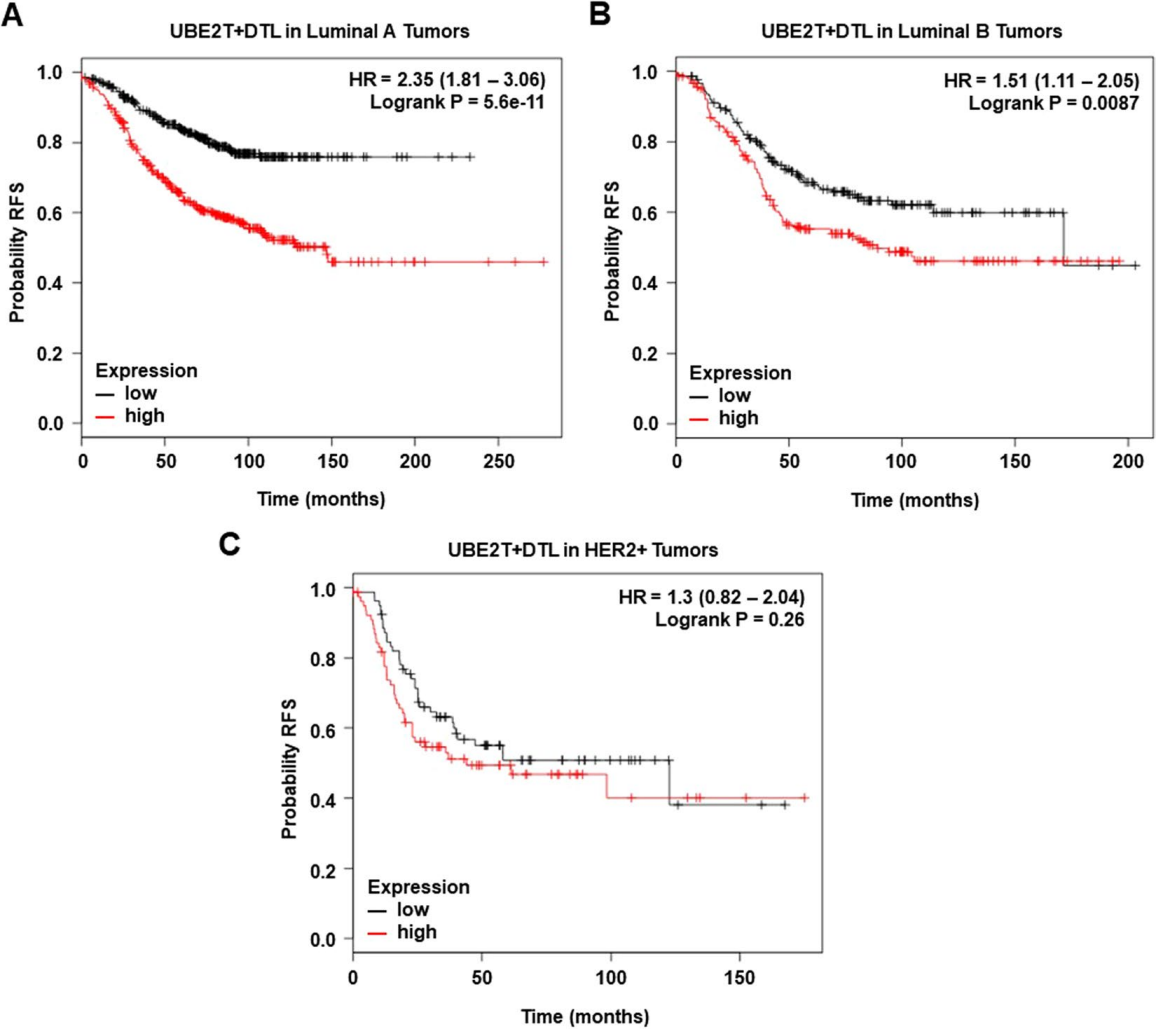
(**A**) Association of the combined analyses of UBE2T and DTL with relapse free survival in Luminal A tumors. (**B**) Association of the combined analyses of UBE2T and DTL with relapse free survival in Luminal B tumors. (**C**) Association of the combined analyses of UBE2T and DTL with relapse free survival in HER2+ tumors.

The combined analyses of UBE2T and *DTL* showed an augmented effect on RFS (Fig. 3B). Similar findings were observed when evaluating outcome only in the treated population (Fig. 3C). In luminal tumors we observed an association with worse outcome in luminal A (Fig. 4A), and B tumors (Fig. 4B), and no association in the HER2 enriched population (Fig. 4C).

Next, we evaluated molecular alterations of these genes at a genomic level in breast cancer patients. To do so, we used data contained at TCGA database as described in material and methods. As can be seen in Table 2, *UBE2T* was amplified in 12% of breast tumors, and *DTL* in 11.9%. Together, these findings suggest that the amplification of these genes contributed to poor prognosis in breast cancer.

**Table 2:** Presence of mutations, amplifications, delections and other alterations in the 29 identified breast cancer genes using data from TCGA, contained at cBioportal.

816 Breast Invasive Carcinoma Samples				
Gene Name	Amplification	Deletion	Mutation	Other alterations
KLHL13. Kelch-like protein 13	0.5%	0.1%	0.5%	—
HUWE1. HECT, UBA and WWE domain containing 1, E3 ubiquitin protein ligase	1.2%	0.4%	3.3%	0.1%
ITCH. Itchy E3 ubiquitin protein ligase	1.8%	0,1%	0.5%	—
KLHDC7A. Kelch domain containing 7A	0.1%	0.7%	0.2%	—
UBE2C. Ubiquitin-conjugating enzyme E2 C	3.7%	0.1%	—	—
UBE2T. Ubiquitin-conjugating enzyme E2 T	12%	—	0.1%	0.1%
UBE2S. Ubiquitin-conjugating enzyme E2 S	2.7%	0.1%	0.1%	—
UBE2H. Ubiquitin-conjugating enzyme E2 H	1%	—	—	—
UBASH3B. Ubiquitin-associated and SH3 domain-containing protein B	—	1.3%	0.1%	—
TRIM71. E3 ubiquitin-protein ligase TRIM71	0.6%	0.2%	0.4%	—
TRIM68. E3 ubiquitin-protein ligase TRIM68	0.2%	0.9%	0.2%	—
TRIM63. E3 ubiquitin-protein ligase TRIM63	0.2%	0.2%	0.1%	—
NEDD4L. E3 ubiquitin-protein ligase NEDD4-like	0.7%	1.1%	0.9%	—
N4BP1. NEDD4-binding protein 1	1.7%	0.6%	0.5%	—
PELI2. E3 ubiquitin-protein ligase pellino homolog 2	0.7%	0.1%	0.5%	—
RNF130. E3 ubiquitin-protein ligase RNF130	1.2%	0.1%	0.1%	—
SOCS2. Suppressor of cytokine signaling 2	1.2%	0.1%	0.1%	—
SIAH1. E3 ubiquitin-protein ligase SIAH1	1.8%	0.7%	0.2%	—
TULP4. Tubby-related protein 4	0.7%	0.6%	0.6%	0.1%
CDCA3. Cell division cycle-associated protein 3	3.4%	0.1%	0.1%	—
CDC20. Cell division cycle-associated protein 20	1.8%	—	0.2%	—
CCDC50. Coiled-coil domain-containing protein 50	2.9%	0.2%	0.5%	—
FBXO32. F-box only protein 32	18.4%	—	—	—
FBXL22. F-box and leucine-rich protein 22	0.6%	0.1%	—	—
FBXO11. F-box only protein 11	0.7%	0.1%	0.6%	—
DTL. Denticleless protein homolog	11.9%	—	0.1%	—
LNX1. E3 ubiquitin-protein ligase LNX	0.7%	—	0.7%	—
SH3RF2. Putative E3 ubiquitin-protein ligase SH3RF2	0.2%	0.1%	0.1%	—
UHRF1. E3 ubiquitin-protein ligase UHRF1	1.5%	0.1%	—	—

### Expression of UBE2T and DTL in other solid tumors

Next, we aimed to evaluate molecular information from other tumor types for which outcome data is available using the online tool KM Plotter, including non-small cell lung adenocarcinoma, ovarian serous cystadenocarcinoma and esophagus-stomach cancer. Of note we did not identify a significant proportion of molecular alterations in those tumors except for non-small cell lung adenocarcinoma patients, where UBE2T was amplified in 7.4% of the tumors, and DTL in 6.5% (Table 3).

**Table 3:** Presence of mutations, amplifications, delections and other alterations in UBE2T and DTL in lung adenocarcinoma, lung squamous cell carcinoma, ovarian cystadenocarcinoma and esophagus-stomach cancer patients using data from TCGA, contained at cBioportal.

Gene Name	Amplification	Deletion	Mutation	Other alterations
**230 Lung Adenocarcinoma Samples**				
UBE2T. Ubiquitin-conjugating enzyme E2T	7,4%	—	0,4%	0,4%
DTL. Denticleless protein homolog	6,5%	—	0,4%	—
**178 Lung Squamous Cell Carcinoma Samples**				
UBE2T. Ubiquitin-conjugating enzyme E2T	—	—	1,1%	—
DTL. Denticleless protein homolog	—	—	1,7%	—
**316 Ovarian Serous Cystadenocarcinoma Samples**				
UBE2T. Ubiquitin-conjugating enzyme E2T	3,2%	—	—	—
DTL. Denticleless protein homolog	0,6%	—	—	—
**265 Esophagus-Stomach Cancer Samples**				
UBE2T. Ubiquitin-conjugating enzyme E2T	2,6%	0,4%	—	—
DTL. Denticleless protein homolog	2,6%	0,8%	—	—

### Association of UBE2T and DTL with outcome in non-small cell lung adenocarcinoma

**Figure 5:**
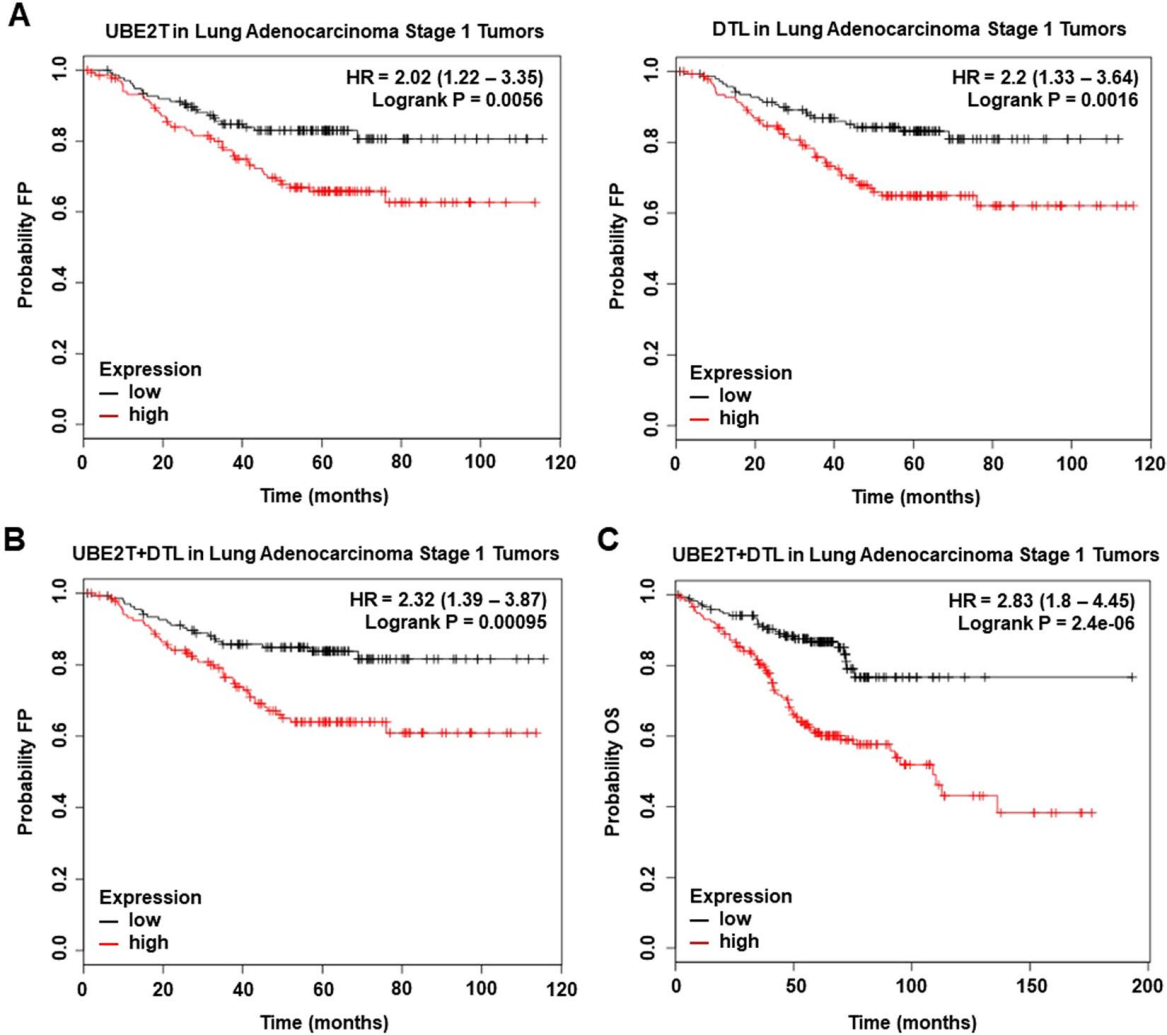
(**A**) Association of UBE2T and DTL individually with first progression survival in stage I Lung Adenocarcinoma tumors. (**B**) Association of the combined analyses of UBE2T and DTL with first progression survival in stage I Lung Adenocarcinoma tumors. (**C**) Association of the combined analyses of UBE2T and DTL with overall survival in stage I Lung Adenocarcinoma tumors.

We studied the association of these two genes with clinical outcome in lung adenocarcinoma as were amplified in more than 6% of patients. Both genes were associated with poor first progression (FP) or relapse (Fig. 5A). The combined expression of *UBE2T* and *DTL* showed a worse effect on FP (Fig. 4B), being of greater magnitude for OS (Fig. 5C).

As described, a clear association with poor outcome was seen only in tumors where amplification was reported. Marginal effects were observed in some tumors (Supplementary Table 2).

## Discussion

In the present article we describe two genes involved in the ubiquitin pathway that are amplified in breast tumors and non-small cell lung adenocarcinomas, and are associated with detrimental outcome. Given the role of this family of proteins and their druggability, these findings have potential for clinical translation.

The protein product of the Ubiquitin-conjugating enzyme *E2T* gene catalyzes the covalent attachment of ubiquitin to protein substrates. It is involved in the DNA repair pathway by catalyzing the monoubiquitination of the Fanconi Anemia Complex^14^ and BRCA1, promoting their degradation^15^. *UBE2T* has been associated with cancer progression and poor outcome in several solid tumors including prostate or gastric cancer^16,17^. In addition, inhibition of UBE2T reduces cell proliferation in osteosarcoma^18^. In breast cancer it has been described that UBE2T has a critical role in the regulation of BRCA1^15^.

DTL mediates the polyubiquitination and subsequent degradation of CDT1, CDKN1A/p21(CIP1) and FBXO18/FBH1. Degradation of p21 permits cells to progress during cell cycle avoiding inhibition at the G1 phase^19^. CDT1 is a critical protein at the G1 phase but also required for the transition along mitosis^20^. FBXO18 is a component of the SCF E3 ubiquitin ligase complex involved in genome maintenance^21^. DTL expression has also been associated with generation of breast cancer in preclinical models, indeed inhibition of this protein reduced cancer growth, through its interaction with Aurora kinase B^22^. Of note, there is limited information about the role of these two proteins in non-small cell lung adenocarcinomas.

Although our article provides novel information we are aware of several limitations. As this is an *in silico* analyses, the findings herewith reported should be confirmed using individual patient samples. In addition, it is unclear if the amplification of these genes in breast cancer is restricted to the basal and luminal subgroup, as data contained at TCGA do not discriminate among subtypes. Finally, the role in cancer of these proteins should be evaluated biochemically and functionally to further characterize the underlying biological mechanism.

Agents targeting components of the ubiquitin pathway are under clinical development. Inhibition of the function of these two proteins could have a potential action against the oncogenic process. Of note, although there are data describing the biological role of *UBE2T* and *DTL* in breast cancer, their association with poor outcome in lung adenocarcinomas has not been described before.

In conclusion, we identified two genes that are amplified in breast tumors and non-small lung adenocarcinomas; and linked with detrimental outcome in the basal and luminal breast subtype and non-small lung adenocarcinomas. Their overexpression facilitates cell cycle progression and avoids DNA repair by inducing the degradation of key regulators of both functions like p21 or BRCA1. The fact that there are efforts to develop agents against components of this pathway makes this finding clinically relevant.

## Material and Methods

### Transcriptomic analysis and identification of upregulated genes

mRNA level data from normal breast tissue and basal-like breast tumors were extracted from a public dataset (GEO DataSet accession number: GDS2250). Affymetrix CEL files were downloaded and analyzed with Affymetrix Transcriptome Analysis Console 3.0. Only genes with minimum 4-fold differential expression values between control and basal-like were selected. To discriminate genes contained at the ubiquitination function we used data contained at Uniprot (www.uniprot.org)^23^. The differentially expressed genes were confirmed independently using Oncomine (www.oncomine.org).

We further performed gene set enrichment analysis (GSEA) to identify Ubiquitin-Proteasome pathway-defined gene sets that varied between normal and Basal-like tissues. Gene sets were collected from the Molecular Signatures Database (MSigDB) (http://www.broadinstitute.org/gsea/msigdb/), including 1 from BioCarta, 2 from Kyoto Encyclopedia of Genes and Genomes (KEGG), and 10 from Gene Ontology (GO) biological process; the data were analyzed by GSEA with parameters set to 1.000 gene-set permutations and gene-sets size between 15 and 500, leaving 12 pathway-defined gene sets in the analysis. The enrichment score corresponds to a weighted Kolmogorov-Smirnov-like statistic and reflects the extent to which the gene set is overrepresented at the extreme (i.e. top or bottom) of the entire ranked list. If the enrichment score is positive (e.g. the gene set is overrepresented by top ranked genes), then the gene set is considered upregulated while it is considered downregulated if the score is negative.

### Outcome analyses

The KM Plotter Online Tool (http://www.kmplot.com)^24,25^ was used to evaluate the relationship between the presence of different genes and patient clinical outcome in different breast cancer subtypes.

This publicly available database allows us to investigate overall survival (OS) and relapse-free survival (RFS) in basal-like, luminal A, luminal B, HER2+ and triple negative breast cancers.

Breast cancer subtypes were defined as follow: Basal-like: ESR1−/HER2−. Luminal A: ESR1+/HER2−/MKI67 low. Luminal B: ESR1+/HER2−/MKI67 high and ESR1+/HER2+. HER2+: ESR1−/HER2+. And Triple negative: ER−/PR−/HER2−.

### Evaluation of molecular alterations

We used data contained at cBioportal (www.cbioportal.org) to explore the presence of mutations, deletions or amplifications in the identified genes^26^.

## Electronic supplementary material

Supplementary Info
