# Supplementary material for: Ubiquitin-conjugating enzyme E2T (UBE2T) and denticleless protein homolog (DTL) are linked to poor outcome in breast and lung cancers
Source: Sci Rep. 2017 Dec 13;7:17530. doi: 10.1038/s41598-017-17836-7 (PMC5727519; doi:10.1038/s41598-017-17836-7)
Supplement: Supplementary file 1 — Supplementary Info [file 41598_2017_17836_MOESM1_ESM.pdf]

Ubiquitin-conjugating enzyme E2T (UBE2T) and denticleless protein homolog (DTL) are linked to poor outcome in breast and lung cancers

Javier Perez-Peña[1], Verónica Corrales-Sánchez[1], Eitan Amir[2], Atanasio Pandiella[3] and Alberto Ocana[1]*

1. Translational Research Unit, Albacete University Hospital and Centro Regional de Investigaciones Biomedicas (CRIB) and CIBERONC, Castilla La Mancha University, Albacete, Spain.
2. Division of Medical Oncology and Hematology, Princess Margaret Cancer Centre, University of Toronto, Toronto, Canada.
3. Cancer Research Center and CIBERONC, CSIC, Salamanca, Spain.

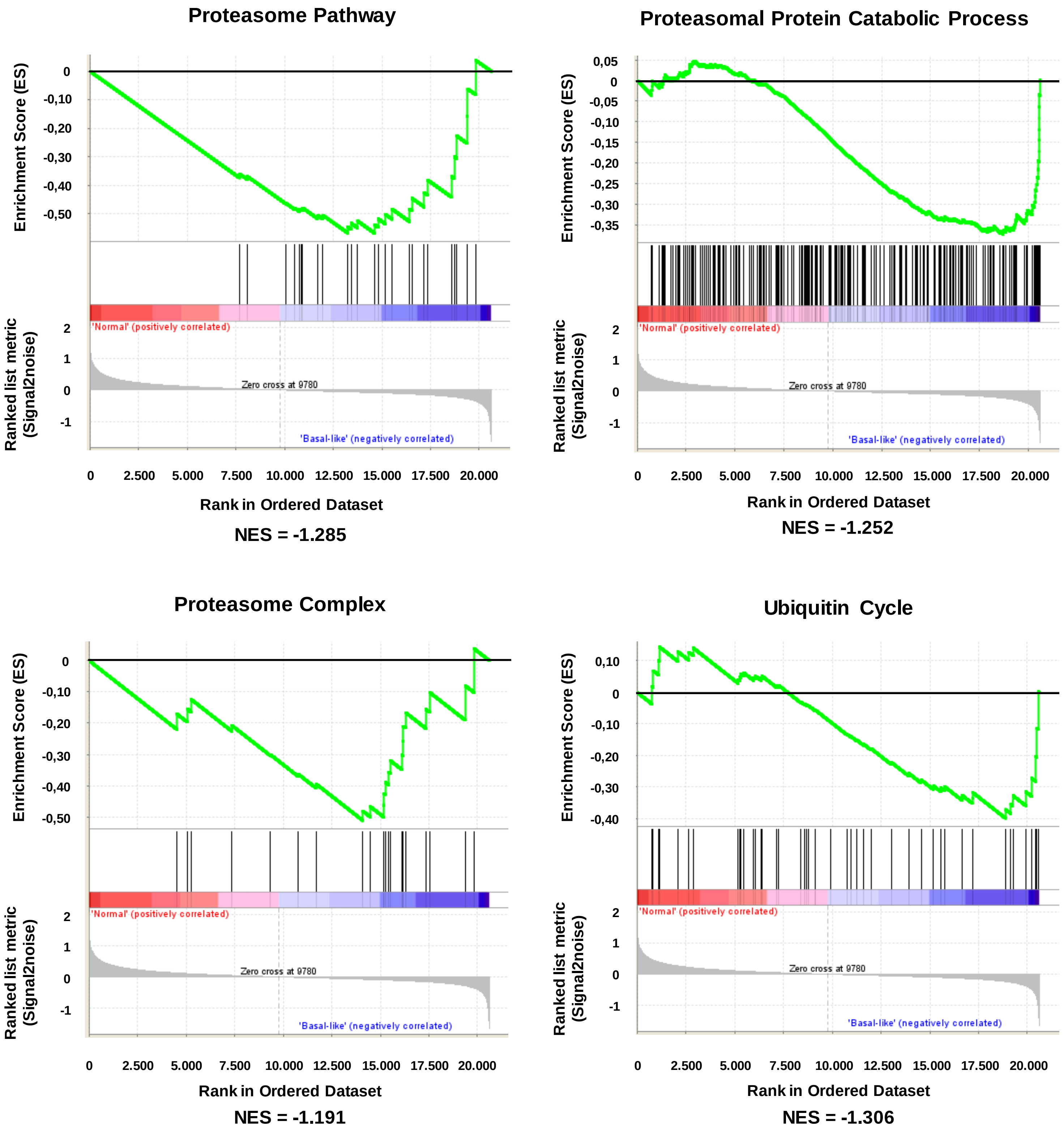

Supplementary Figure 1

Gene name	Gene symbol	Function	
HECT, UBA and WWE domain containing 1, E3 ubiquitin protein ligase	HUWE1	E3 ubiquitin-protein ligase which mediates ubiquitination and subsequent proteasomal degradation of target proteins. Regulates apoptosis by catalyzing the polyubiquitination and degradation of MCL1. Mediates monoubiquitination of DNA polymerase beta (POLB) at 'Lys-41', 'Lys-61' and 'Lys-81', thereby playing a role in base-excision repair. Also ubiquitinates the p53/TP53 tumor suppressor and core histones including H1, H2A, H2B, H3 and H4. Binds to an upstream initiator-like sequence in the preprodynorphin gene. May regulate abundance of CDC6 after DNA damage by polyubiquitinating and targeting CDC6 to degradation. Mediates polyubiquitination of isoform 2 of PA2G4.	Apoptosis / DNA Damage
E3 ubiquitin-protein ligase Itchy homolog	ITCH	Acts as an E3 ubiquitin-protein ligase which accepts ubiquitin from an E2 ubiquitin-conjugating enzyme in the form of a thioester and then directly transfers the ubiquitin to targeted substrates. It catalyzes 'Lys-29'-, 'Lys-48'- and 'Lys-63'-linked ubiquitin conjugation. It is involved in the control of inflammatory signaling pathways. Is an essential component of a ubiquitin-editing protein complex, comprising also TNFAIP3, TAX1BP1 and RNF11, that ensures the transient nature of inflammatory signaling pathways. Regulates the transcriptional activity of several transcription factors, and probably plays an important role in the regulation of immune response. Involved in the regulation of apoptosis and reactive oxygen species levels through the ubiquitination and proteasomal degradation of TXNIP. Mediates the antiapoptotic activity of epidermal growth factor through the ubiquitination and proteasomal degradation of p15 BID. Overexpression delays G2/M phase progression.	Inflammatory Signaling / Immune Response / Apoptosis /Cell Cycle
Ubiquitin-conjugating enzyme E2 C	UBE2C	Accepts ubiquitin from the E1 complex and catalyzes its covalent attachment to other proteins. In vitro catalyzes 'Lys-11'- and 'Lys-48'-linked polyubiquitination. Acts as an essential factor of the anaphase promoting complex/cyclosome (APC/C), a cell cycle-regulated ubiquitin ligase that controls progression through mitosis. Acts by initiating 'Lys-11'-linked polyubiquitin chains on APC/C substrates, leading to the degradation of APC/C substrates by the proteasome and promoting mitotic exit.	Cell Cycle
Ubiquitin-conjugating enzyme E2 S	UBE2S	Accepts ubiquitin from the E1 complex and catalyzes its covalent attachment to other proteins. Catalyzes 'Lys-11'-linked polyubiquitination. Acts as an essential factor of the anaphase promoting complex/cyclosome (APC/C), a cell cycle-regulated ubiquitin ligase that controls progression through mitosis. Acts by specifically elongating 'Lys-11'-linked polyubiquitin chains initiated by the E2 enzyme UBE2C/UBCH10 on APC/C substrates, enhancing the degradation of APC/C substrates by the proteasome and promoting mitotic exit. Also acts by elongating ubiquitin chains initiated by the E2 enzyme UBE2D1/UBCH5 in vitro; it is however unclear whether UBE2D1/UBCH5 acts as an E2 enzyme for the APC/C in vivo. Also involved in ubiquitination and subsequent degradation of VHL, resulting in an accumulation of HIF1A. In vitro able to promote polyubiquitination using all 7 ubiquitin Lys residues, except 'Lys-48'-linked polyubiquitination.	Cell Cycle
Ubiquitin-conjugating enzyme E2 S	UBE2H	Accepts ubiquitin from the E1 complex and catalyzes its covalent attachment to other proteins. In vitro catalyzes 'Lys-11'- and 'Lys-48'-linked polyubiquitination. Capable, in vitro, to ubiquitinate histone H2A.	-
Ubiquitin-associated and SH3 domain-containing protein B	UBASH3B	Interferes with CBL-mediated down-regulation and degradation of receptor-type tyrosine kinases. Promotes accumulation of activated target receptors, such as T-cell receptors and EGFR, on the cell surface. Exhibits tyrosine phosphatase activity toward several substrates including EGFR, FAK, SYK, and ZAP70. Down-regulates proteins that are dually modified by both protein tyrosine phosphorylation and ubiquitination.	Cell Proliferation / Immune Response
E3 ubiquitin-protein ligase TRIM71	TRIM71	E3 ubiquitin-protein ligase that cooperates with the microRNAs (miRNAs) machinery and promotes embryonic stem cells proliferation and maintenance. Binds to miRNAs and associates with AGO2, participating in post-transcriptional repression of transcripts such as CDKN1A. Facilitates the G1-S transition to promote rapid embryonic stem cell self-renewal by repressing CDKN1A expression.	Cell Proliferation / Cell Cycle
NEDD4-binding protein 1	N4BP1	Inhibitor of the E3 ubiquitin-protein ligase ITCH. Acts by interacting with the second WW domain of ITCH, leading to compete with ITCH's substrates and impairing ubiquitination of substrates (By similarity).	Inflammatory / Immune Response / Apoptosis /Cell Cycle
E3 ubiquitin-protein ligase RNF130	RNF130	May have a role during the programmed cell death of hematopoietic cells (By similarity). Acts as an E3 ubiquitin-protein ligase.	Cell Death
Tubby-related protein 4	TULP4	May be a substrate-recognition component of a SCF-like ECS (Elongin-Cullin-SOCS-box protein) E3 ubiquitin ligase complex which mediates the ubiquitination and subsequent proteasomal degradation of target proteins.	-
Cell division cycle-associated protein 3	CDCA3	F-box-like protein which is required for entry into mitosis. Acts by participating in E3 ligase complexes that mediate the ubiquitination and degradation of WEE1 kinase at G2/M phase (By similarity).	Cell Cycle
Cell division cycle-associated protein 20	CDC20	Activator protein that regulates the ubiquitin ligase activity and substrate specificity of the anaphase promoting complex/cyclosome (APC/C). At the metaphase-to-anaphase transition, recognizes and binds proteins containing a D-box including the B-type cyclins CLB2 and CLB5, HSL1 and securin PDS1, and recruits them in a C-box-dependent manner to the APC/C for ubiquitination and subsequent proteolysis. Required for sister chromatid separation and disassembly of the mitotic spindle. Target of the spindle checkpoint pathway through participation in the mitotic checkpoint complex (MCC) and the MAD2-CDC20 subcomplex. MCC and presumably the MAD2-CDC20 subcomplex inhibit the ubiquitin ligase activity of the anaphase promoting complex/cyclosome (APC/C) by preventing its activation by CDC20 until proper attachment of all chromosomes to the spindle.	Cell Cycle
F-box only protein 11	FBXO11	Substrate recognition component of a SCF (SKP1-CUL1-F-box protein) E3 ubiquitin-protein ligase complex which mediates the ubiquitination and subsequent proteasomal degradation of target proteins, such as DTL/CDT2, BCL6 and PRDM1/BLIMP1. The SCF(FBXO11) complex mediates ubiquitination and degradation of BCL6, thereby playing a role in the germinal center B-cells terminal differentiation toward memory B-cells and plasma cells. The SCF(FBXO11) complex also mediates ubiquitination and degradation of DTL, an important step for the regulation of TGF-beta signaling, cell migration and the timing of the cell-cycle progression and exit. Binds to and neddylates phosphorylated p53/TP53, inhibiting its transcriptional activity. SCF(FBXO11) does not seem to direct ubiquitination of p53/TP53.	Cell Death / Cell Proliferation / Cell Migration / Cell Cycle
E3 ubiquitin-protein ligase UHRF1	UHRF1	Multidomain protein that acts as a key epigenetic regulator by bridging DNA methylation and chromatin modification. Specifically recognizes and binds hemimethylated DNA at replication forks via its YDG domain and recruits DNMT1 methyltransferase to ensure faithful propagation of the DNA methylation patterns through DNA replication. In addition to its role in maintenance of DNA methylation, also plays a key role in chromatin modification: through its tudor-like regions and PHD-type zinc fingers, specifically recognizes and binds histone H3 trimethylated at 'Lys-9' (H3K9me3) and unmethylated at 'Arg-2' (H3R2me0), respectively, and recruits chromatin proteins. Enriched in pericentric heterochromatin where it recruits different chromatin modifiers required for this chromatin replication. Also localizes to euchromatic regions where it negatively regulates transcription possibly by impacting DNA methylation and histone modifications. Has E3 ubiquitin-protein ligase activity by mediating the ubiquitination of target proteins such as histone H3 and PML. It is still unclear how E3 ubiquitin-protein ligase activity is related to its role in chromatin in vivo. May be involved in DNA repair.	Epigenetic Regulation / DNA Damage

Supplementary Table 1

		Lung adenocarcinoma Stage 1 patients						Lung squamous cell carcinoma Stage 1 patients					
		First Progression (FP)			Overall Survival (OS)			First Progression (FP)			Overall Survival (OS)		
**Gene name**	**Protein name**	**HR**	**p-value**	**q-value**	**HR**	**p-value**	**q-value**	**HR**	**p-value**	**q-value**	**HR**	**p-value**	**q-value**
UBE2T	Ubiquitin-conjugating enzyme E2 T	2,02 (1,22 - 3,35)	5,60E-03	0,013	3,48 (2,16 - 5,61)	5,00E-08	3,42E-07	0,68 (0,19 - 2,4)	0,54	0,26	1,78 (0,97 - 3,27)	0,06	0,041
DTL	Denticleless protein homolog	2,2 (1,33 - 3,64)	1,60E-03	5,48E-03	1,65 (1,11 - 2,46)	0,013	0,022	0,99 (0,28 - 3,49)	0,99	0,42	1,1 (0,72 - 1,68)	0,66	0,33

		Ovarian cystadenocarcinoma Stage 1+2 patients					
		Progression Free Survival (PFS)			Overall Survival (OS)		
**Gene name**	**Protein name**	**HR**	**p-value**	**q-value**	**HR**	**p-value**	**q-value**
UBE2T	Ubiquitin-conjugating enzyme E2 T	1,58 (0,75 - 3,33)	0,22	0,11	1,94 (0,66 - 5,7)	0,22	0,11
DTL	Denticleless protein homolog	1,89 (1,06 - 3,38)	0,029	0,027	2,5 (1,09 - 5,76)	0,026	0,027

		Gastric cancer Stage 2 patients					
		First Progression (FP)			Overall Survival (OS)		
**Gene name**	**Protein name**	**HR**	**p-value**	**q-value**	**HR**	**p-value**	**q-value**
UBE2T	Ubiquitin-conjugating enzyme E2 T	0,57 (0,31 - 1,06)	0,073	0,045	0,54 (0,28 - 1,03)	0,058	0,041
DTL	Denticleless protein homolog	0,52 (0,28 - 0,95)	0,031	0,027	0,49 (0,27 - 0,91)	0,021	0,027

Supplementary Table 2

Supplementary Figure 1. Enrichment plots of the identified genes, using different gene sets showing the upregulation of the ubiquitin pathway in Basal-like breast tumors, represented by the Normalized Enrichment Score (NES). Only gene sets at FDR <0.25 were considered significantly enriched.

Supplementary Table 1. Biological functions of the 16 identified upregulated genes.

Supplementary Table 2. Association of UBE2T and DTL with first progression and overall survival in patients with lung adenocarcinoma, lung squamous cell carcinoma, ovarian cystadenocarcinoma, and esophagus-stomach cancer.
